# Supplementary material for: Associations between the Number of Children, Depressive Symptoms, and Cognition in Middle-Aged and Older Adults: Evidence from the China Health and Retirement Longitudinal Study
Source: Healthcare (Basel). 2024 Sep 26;12(19):1928. doi: 10.3390/healthcare12191928 (PMC11475735; doi:10.3390/healthcare12191928)
Supplement: Supplementary file 1 [file healthcare-12-01928-s001.zip › healthcare-3218086-supplementary.pdf]

Supplementary Table S1. Variable Selection and Definition

	Name of Variables	Variable Assignment		Name of Variables	Variable Assignment
1	Age [a]	Continuous variable in years.	12	Smoking	0 = No<br>1 = Yes
2	Gender	0 = Female<br>1 = Male	13	Drinking	0 = No<br>1 = Yes
3	Residence	0 = Rural<br>1 = Urban	14	Hypertension	0 = No<br>1 = Yes
4	Education level	0 = Below Primary School<br>1 = Primary School<br>2 = Middle School<br>3 = High School and Above	15	Diabetes	0 = No<br>1 = Yes
5	Marital status	0 = Divorced/Separated/Widowed/Never Married<br>1 = Married/Cohabitated	16	Lung Disease	0 = No<br>1 = Yes
6	Total Cognition [b]	Continuous variable in scores with a range of [0, 21]. Higher scores indicate higher levels of cognitive function.	17	Stroke	0 = No<br>1 = Yes
7	Episodic Memory [c]	Continuous variable in scores with a range of [0, 10]. Higher scores indicate higher levels of episodic memory.	18	Mental Disorder	0 = No<br>1 = Yes
8	Mental Status [d]	Continuous variable in scores with a range of [0, 11]. Higher scores indicate higher levels of mental status.			
9	CES-D10 [e]	Continuous variable in scores with a range of [0, 30]. With a score of 10 or above indicating the presence of depressive symptoms.	19	Heart Disease	0 = No<br>1 = Yes
10	Sleep Duration (h) [f]	0 = (0, 6]<br>1 = (6, 8]<br>2 = ＞8	20	Liver Disease	0 = No<br>1 = Yes
11	Life Satisfaction [g]	1 = Very Good<br>2 = Good<br>3 = Fair<br>4 = Poor<br>5 = Very poor	21	Kidney Disease	0 = No<br>1 = Yes

[a]: The real age is the interview year minus the respondent's birth year. [b]: Summed scores from episodic memory and mental status assessments. Derived from TICS measures: self-rated memory, date, day, season; recall and delayed recall of 10 words; serial subtraction from 100 by sevens; and drawing overlapping pentagons. [c]: Reflecting respondents' immediate and delayed recall. [d]: This encompasses general cognitive functions such as orientation (e.g., knowing the current date, day of the week, and season), calculation abilities (e.g., serial subtraction tasks), and visuospatial skills (e.g., drawing overlapping geometric figures). e: Measured depressive symptoms using the 10-item Centre for Epidemiologic Studies Depression Scale. f: During the past month, how many hours of actual sleep did you get at night? Average hours for one night. g: How satisfied are you with your life overall? Are you felt Very Good, Good, Fair, Poor, or Very poor

Supplementary Table S2. Associations Between Number of Children and Depression in older adults Based on Cross-sectional Study Data

Models	Depression	
	β	*95%CI*
**Crude model**		
**Number of Children**		
1	Ref.	-
0	0.670**	(0.234,1.106)
2	0.349***	(0.184,0.514)
3	0.422***	(0.248,0.597)
≥4	0.509***	(0.330,0.689)
**Model 1**		
**Number of Children**		
1	Ref.	-
0	0.614**	(0.233,0.995)
2	0.201*	(0.048,0.354)
3	0.233**	(0.065,0.400)
≥4	0.320***	(0.137,0.503)
**Model 2**		
**Number of Children**		
1	Ref.	-
0	0.561**	(0.168,0.963)
2	0.212**	(0.079,0.346)
3	0.235***	(0.091,0.380)
≥4	0.321***	(0.162,0.479)

Crude model: No adjustments. Model 1: Partially adjusted model, adjusting for age, sex, residence, education level, and marital status. Model 2: Fully adjusted model, additionally adjusting for smoking, drinking, life satisfaction, sleep duration, hypertension, diabetes, lung disease, stroke, mental disorder, heart disease, liver disease, and kidney disease. Ref: One child was chosen as the reference value, considering that the participants were affected by the one-child policy in China. *$P < 0.05$, **$P < 0.01$, ***$P < 0.001$.

Supplementary Table S3. Comparing the models

Model	AIC	BIC
Crude Model	29942.30	29974.69
Model 2: Fixed effects	28814.79	28937.87
Model 3: Full	28352.44	28643.96

Supplementary Table S4. Associations Between Number of Children, Depression, and Cognitive Function in older adults Based on Cross-sectional Study Data Using Multiple Linear Regression Model

Models	Episodic Memory		Mental Status		Total Cognition	
	β	*95%CI*	*β*	*95%CI*	*β*	*95%CI*
**Crude model**						
**Number of Children**						
1	Ref.	-	Ref.	-	Ref.	-
0	-0.687*	(-1.271,-0.102)	-0.835*	(-1.561,-0.110)	-1.522**	(-2.586,-0.459)
2	-0.324***	(-0.453,-0.195)	-0.526***	(-0.686,-0.366)	-0.850***	(-1.085,-0.615)
3	-0.590***	(-0.736,-0.443)	-0.759***	(-0.941,-0.578)	-1.349***	(-1.615,-1.083)
≥4	-1.003***	(-1.164,-0.841)	-1.029***	(-1.229,-0.828)	-2.031***	(-2.325,-1.737)
**Depression**						
0	Ref.	-	Ref.	-	Ref.	-
1	-0.626***	(-0.735,-0.518)	-0.817***	(-0.951,0.683)	-1.443***	(-1.640,-1.246)
**Model 1**						
**Number of Children**						
1	Ref.	-	Ref.	-	Ref.	-
0	-0.306	(-0.855,0.244)	-0.471	(-1.142,0.201)	-0.776	(-1.738,0.186)
2	-0.038	(-0.163,0.087)	-0.184*	(-0.336,-0.031)	-0.221*	(-0.440,-0.003)
3	-0.080	(-0.226,0.065)	-0.275**	(-0.453,-0.097)	-0.356**	(-0.611,-0.100)
≥4	-0.211*	(-0.381,-0.042)	-0.395***	(-0.602,-0.187)	-0.606***	(-0.903,-0.309)
**Depression**						
0	Ref.	-	Ref.	-	Ref.	-
1	-0.484***	(-0.587,-0.382)	-0.489***	(-0.615,-0.363)	-0.973***	(-1.153,-0.794)
**Model 2**						
**Number of Children**						
1	Ref.	-	Ref.	-	Ref.	-
0	-0.330	(-0.877,0.216)	-0.497	(-1.165,0.171)	-0.827	(-1.781,0.127)
2	-0.033	(-0.157,0.092)	-0.170*	(-0.322,-0.018)	-0.203	(-0.420,0.014)
3	-0.067	(-0.213,0.078)	-0.247**	(-0.424,-0.069)	-0.314*	(-0.568,-0.061)
≥4	-0.192*	(-0.361,-0.024)	-0.355***	(-0.562,-0.149)	-0.548***	(-0.843,-0.253)
**Depression**						
0	Ref.	-	Ref.	-	Ref.	-
1	-0.461***	(-0.570,-0.353)	-0.505***	(-0.638,-0.372)	-0.966***	(-1.155,-0.778)

Crude model: No adjustments. Model 1: Partially adjusted model, adjusting for age, sex, residence, education level, and marital status. Model 2: Fully adjusted model, additionally adjusting for smoking, drinking, life satisfaction, sleep duration, hypertension, diabetes, lung disease, stroke, mental disorder, heart disease, liver disease, and kidney disease. Ref: One child was chosen as the reference value, considering that the participants were affected by the one-child policy in China. *$P < 0.05$, **$P < 0.01$, ***$P < 0.001$.

Supplementary Table S5. Relationship Between the Number of Children and Cognitive Function mediated by Depressive Symptoms Using Multiple Linear Regression Model

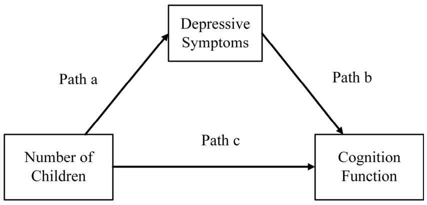

Path a: Exposure predicting mediator
Path b: Mediator predicting outcome
Path c: Exposure predicting outcome

Models	Crude Model	Model 1	Model 2
**Coefficients (95% CI)**			
Path a	0.035*** (0.022， 0.047)	0.020** (0.001,0.034)	0.020** (0.007,0.034)
Path b	-1.336*** (-1.531,-1.142)	-0.957*** (-1.138,-0.776)	-0.993*** (-1.181,0.805)
Path c	-0.613*** (-0.701,-0.524)	-0.168*** (-0.259,-0.078)	-0.161*** (-0.251,-0.071)
**Effect**			
Total effect [a]	-0.566*** (-0.653,-0.479)	-0.149*** (-0.239,-0.060)	-0.993*** (-1.181.-0.805)
Indirect effect [b]	-0.046***	-0.019**	-0.020**
pMe [c]	8.20%	12.80%	14.23%

Crude model: No adjustments. Model 1: Partially adjusted model, adjusting for age, sex, residence, education level, and marital status. Model 2: Fully adjusted model, additionally adjusting for smoking, drinking, life satisfaction, sleep duration, hypertension, diabetes, lung disease, stroke, mental disorder, heart disease, liver disease, and kidney disease. [a] Total effects represent the overall impact of the number of children and depression on cognitive functioning, expressed through regression coefficients with 95% confidence intervals (CIs). [b] Indirect effect = Multiplication of Path a and Path b coefficients. Coefficients for indirect effects were determined using the Sobel-Goodman Mediation Test. [C] pMe is the proportion of the total effect that is mediated. *P < 0.05, **P < 0.01, ***P < 0.001.

Supplementary Table S6. Associations Between Number of Children, Depression, and Cognitive Function in older adults (Grouped)

Models	Episodic Memory		Mental Status		Total Cognition	
	β	*95%CI*	*β*	*95%CI*	*β*	*95%CI*
**Male**						
**Number of Children**						
1	Ref.	-	Ref.	-	Ref.	-
0	-0.003	(-0.155,0.150)	-0.059	(-0.158,0.040)	-0.040	(-0.132,0.053)
2	0.008	(-0.028,0.045)	-0.022	(-0.045,0.001)	-0.010	(-0.032,0.011)
3	-0.002	(-0.045,0.040)	-0.030*	(-0.056,-0.003)	-0.019	(-0.045,0.006)
≥4	-0.017	(-0.069,0.034)	-0.023	(-0.054,0.008)	-0.020	(-0.050,0.010)
**Depression**						
0	Ref.	-	Ref.	-	Ref.	-
1	-0.103&&&	(-0.140,-0.066)	-0.064***	(-0.087,-0.042)	-0.078***	(-0.099,-0.057)
**Female**						
**Number of Children**						
1	Ref.	-	Ref.	-	Ref.	-
0	-0.165	(-0.372,0.043)	-0.084	(-0.234,0.067)	-0.113	(-0.248,0.022)
2	-0.022	(-0.058,0.013)	-0.010	(-0.037,0.018)	-0.014	(-0.038,0.010)
3	-0.027	(-0.071,0.018)	-0.021	(-0.056,0.014)	-0.023	(-0.053,0.008)
≥4	-0.092**	(-0.148,-0.035)	-0.076***	(-0.120,-0.032)	-0.082***	(-0.120,-0.043)
**Depression**						
0	Ref.	-	Ref.	-	Ref.	-
1	-0.100***	(-0.133,-0.066)	-0.065***	(-0.091,-0.038)	-0.078***	(-0.100,-0.055)
**Urban**						
**Number of Children**						
1	Ref.	-	Ref.	-	Ref.	-
0	-0.034	(-0.176,0.109)	-0.014	(-0.106,0.078)	-0.021	(-0.109,0.066)
2	0.006	(-0.025,0.038)	-0.009	(-0.030,0.013)	-0.003	(-0.023,0.017)
3	-0.018	(-0.061,0.025)	-0.031*	(-0.060,-0.003)	-0.026	(-0.052,0.001)
≥4	-0.083**	(-0.140,-0.026)	-0.046*	(-0.082,-0.011)	-0.058***	(-0.092,-0.024)
**Depression**						
0	Ref.	-	Ref.	-	Ref.	-
1	-0.116***	(-0.154,-0.078)	-0.054***	(-0.078,-0.029)	-0.076***	(-0.100,-0.053)
**Rural**						
**Number of Children**						
1	Ref.	-	Ref.	-	Ref.	-
0	-0.132	(-0.357,0.093)	-0.165*	(-0.330,-0.001)	-0.152*	(-0.298,-0.007)
2	-0.037	(-0.080,0.005)	-0.032*	(-0.063,-0.001)	-0.033*	(-0.061,-0.005)
3	-0.030	(-0.077,0.017)	-0.033	(-0.067,0.001)	-0.031*	(-0.062,-0.001)
≥4	-0.044	(-0.099,0.010)	-0.048*	(-0.086,-0.009)	-0.046*	(-0.080,-0.011)
**Depression**						
0	Ref.	-	Ref.	-	Ref.	-
1	-0.094***	(-0.127,-0.061)	-0.071***	(-0.095,-0.048)	-0.080***	(-0.101,-0.059)

Fully adjusted models are used. Ref: One child was chosen as the reference value, considering that the participants were affected by the one-child policy in China. *$P < 0.05$, **$P < 0.01$, ***$P < 0.001$

Supplementary Table S7. Relationship Between the Number of Children and Cognitive Function mediated by Depressive Symptoms (Male)

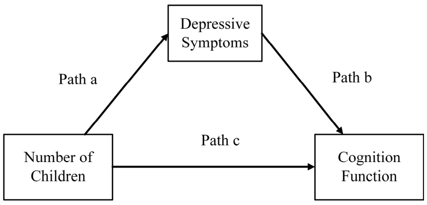

Path a: Exposure predicting mediator
Path b: Mediator predicting outcome
Path c: Exposure predicting outcome

Models	Crude Model	Model 1	Model 2
**Coefficients (95% CI)**			
Path a	0.027** (0.012,0.043)	0.009 (-0.009,0.026)	0.011 (-0.005,0.028)
Path b	-1.396*** (-1.663,-1.128)	-1.028*** (-1.279,-0.778)	-0.978*** (-1.235,-0.721)
Path c	-0.040*** (-0.518,-0.292)	-0.083 (-0.199,0.033)	-0.073 (-0.188,0.042)
**Effect**			
Total effect [a]	-0.367*** (-0.478,-0.256)	-0.074 (-0.189,0.040)	-0.062 (-0.176,0.052)
Indirect effect [b]	-0.038** (-0.060,-0.016)	-0.009 (-0.026,-0.009)	-0.011 (-0.029,0.005)
pMe [c]	10.35%	12.16%	17.74%

[a] Total effects represent the overall impact of the number of children and depression on cognitive functioning, expressed through regression coefficients with 95% confidence intervals (CIs). [b] Indirect effect = Multiplication of Path a and Path b coefficients. Coefficients for indirect effects were determined using the bootstrap method with 1000 repetitions, and a 95% bias-corrected and accelerated (BCa) confidence interval (CI) was employed to enhance the accuracy of the analysis. [C] pMe is the proportion of the total effect that is mediated. *P < 0.05, **P < 0.01, ***P < 0.001.

Supplementary Table S8. Relationship Between the Number of Children and Cognitive Function mediated by Depressive Symptoms (Female)

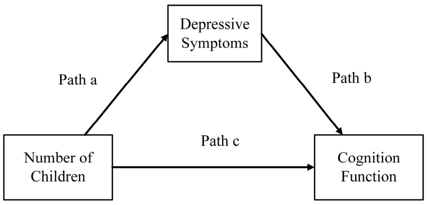

Path a: Exposure predicting mediator
Path b: Mediator predicting outcome
Path c: Exposure predicting outcome

Models	Crude Model	Model 1	Model 2
**Coefficients (95% CI)**			
Path a	0.052*** (0.031,0.072)	0.037** (0.013,0.060)	0.029** (0.007,0.051)
Path b	-1.195*** (-1.483,-0.906)	-0.887*** (-1.146,-0.628)	-0.976*** (-1.250,-0.703)
Path c	-0.914*** (-1.055,-0.774)	-0.274*** (-0.417,-0.132)	-0.265*** (-0.407,-0.123)
**Effect**			
Total effect [a]	-0.852*** (-0.099,-0.714)	-0.242** (-0.383,-0.101)	-0.236** (-0.377,-0.096)
Indirect effect [b]	-0.062*** (-0.096,-0.037)	-0.032** (-0.059,-0.012)	-0.028* (-0.058,-0.007)
pMe [c]	7.28%	13.22%	11.86%

[a] Total effects represent the overall impact of the number of children and depression on cognitive functioning, expressed through regression coefficients with 95% confidence intervals (CIs). [b] Indirect effect = Multiplication of Path a and Path b coefficients. Coefficients for indirect effects were determined using the bootstrap method with 1000 repetitions, and a 95% bias-corrected and accelerated (BCa) confidence interval (CI) was employed to enhance the accuracy of the analysis. [C] pMe is the proportion of the total effect that is mediated. *P < 0.05, **P < 0.01, ***P < 0.001.

Supplementary Table S9. Relationship Between the Number of Children and Cognitive Function mediated by Depressive Symptoms (Urban)

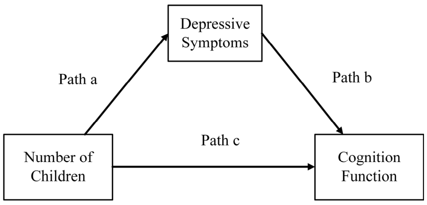

Path a: Exposure predicting mediator
Path b: Mediator predicting outcome
Path c: Exposure predicting outcome

Models	Crude Model	Model 1	Model 2
**Coefficients (95% CI)**			
Path a	0.039*** (0.022,0.056)	0.035*** (0.016,0.054)	0.035*** (0.016,0.053)
Path b	-1.253*** (-1.555,-0.951)	-1.002*** (-1.282,-0.722)	-1.028*** (-1.318,-0.737)
Path c	-0.683*** (-0.808,-0.557)	-0.239*** (-0.368,-0.111)	-0.223** (-0.351,-0.095)
**Effect**			
Total effect [a]	-0.634*** (-0.758,-0.510)	-0.204** (-0.332,-0.077)	-0.188** (-0.315,-0.061)
Indirect effect [b]	-0.049*** (-0.076,-0.026)	-0.035** (-0.060,-0.018)	-0.035** (-0.057,-0.017)
pMe [c]	7.73%	17.16%	18.62%

[a] Total effects represent the overall impact of the number of children and depression on cognitive functioning, expressed through regression coefficients with 95% confidence intervals (CIs). [b] Indirect effect = Multiplication of Path a and Path b coefficients. Coefficients for indirect effects were determined using the bootstrap method with 1000 repetitions, and a 95% bias-corrected and accelerated (BCa) confidence interval (CI) was employed to enhance the accuracy of the analysis. [C] pMe is the proportion of the total effect that is mediated. *P < 0.05, **P < 0.01, ***P < 0.001.

Supplementary Table S10. Relationship Between the Number of Children and Cognitive Function mediated by Depressive Symptoms (Rural)

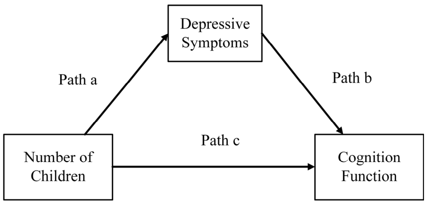

Path a: Exposure predicting mediator
Path b: Mediator predicting outcome
Path c: Exposure predicting outcome

Models	Crude Model	Model 1	Model 2
**Coefficients (95% CI)**			
Path a	0.012 (-0.006,0.031)	0.008 (-0.012,0.028)	0.008 (-0.011,0.027)
Path b	-1.245*** (-1.499,-0.990)	-0.928*** (-1.163,-0.692)	-0.952*** (-1.197,-0.706)
Path c	-0.372*** (-0.499,-0.244)	-0.112 (-0.238,0.014)	-0.112 (-0.238,0.013)
**Effect**			
Total effect [a]	-0.356*** (-0.481,-0.231)	-0.104 (-0.229,0.021)	-0.105 (-0.229,0.020)
Indirect effect [b]	-0.015 (-0.038,0.009)	-0.008 (-0.027,0.012)	-0.008 (-0.028,0.010)
pMe [c]	4.21%	7.69%	7.62%

[a] Total effects represent the overall impact of the number of children and depression on cognitive functioning, expressed through regression coefficients with 95% confidence intervals (CIs). [b] Indirect effect = Multiplication of Path a and Path b coefficients. Coefficients for indirect effects were determined using the bootstrap method with 1000 repetitions, and a 95% bias-corrected and accelerated (BCa) confidence interval (CI) was employed to enhance the accuracy of the analysis. [C] pMe is the proportion of the total effect that is mediated. *P < 0.05, **P < 0.01, ***P < 0.001.

Supplementary Table S11. Relationship Between the Number of Children and Cognitive Function mediated by Depressive Symptoms (No grouping of the number of children)

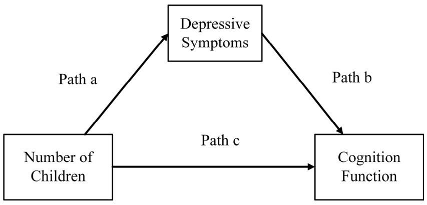

Path a: Exposure predicting mediator
Path b: Mediator predicting outcome
Path c: Exposure predicting outcome

Models	Crude Model	Model 1	Model 2
**Coefficients (95% CI)**			
Path a	0.026*** (0.016,0.037)	0.023*** (0.012,0.035)	0.024*** (0.013,0.035)
Path b	-1.344*** (-1.539,-1.149)	-0.956*** (-1.135,-0.776)	-0.972*** (-1.159,-0.047)
Path c	-0.490*** (-0.562,-0.419)	-0.145*** (-0.218,-0.071)	-0.134*** (-0.207,-0.062)
**Effect**			
Total effect [a]	-0.455*** (-0.526,-0.384)	-0.130*** (-0.203,-0.577)	-0.119*** (-0.192,-0.047)
Indirect effect [b]	-0.035*** (-0.051,-0.021)	-0.022*** (-0.035,-0.012)	-0.017** (-0.032,-0.005)
pMe [c]	7.69%	16.92%	14.29%

[a] Total effects represent the overall impact of the number of children and depression on cognitive functioning, expressed through regression coefficients with 95% confidence intervals (CIs). [b] Indirect effect = Multiplication of Path a and Path b coefficients. Coefficients for indirect effects were determined using the bootstrap method with 1000 repetitions, and a 95% bias-corrected and accelerated (BCa) confidence interval (CI) was employed to enhance the accuracy of the analysis. [C] pMe is the proportion of the total effect that is mediated. *P < 0.05, **P < 0.01, ***P < 0.001.

Supplementary Table S12. Relationship Between the Number of Children and Cognitive Function mediated by Depressive Symptoms (Regrouped)

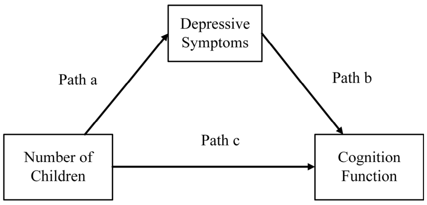

Path a: Exposure predicting mediator
Path b: Mediator predicting outcome
Path c: Exposure predicting outcome

Models	Crude Model	Model 1	Model 2
**Coefficients (95% CI)**			
Path a	0.035*** (0.022,0.047)	0.020** (0.006,0.034)	0.019** (0.006,0.033)
Path b	-1.336*** (-1.531,-1.142)	-0.955*** (-1.135,-0.773)	-0.972*** (-1.159,-0.786)
Path c	-0.613*** (-0.701,-0.524)	-0.171*** (-0.261,-0.081)	-0.159*** (-0.248,-0.069)
**Effect**			
Total effect [a]	-0.566*** (-0.653,-0.479)	-0.152*** (-0.241,-0.063)	-0.140** (-0.228,-0.051)
Indirect effect [b]	-0.046*** (-0.065,-0.029)	-0.019** (-0.034,-0.005)	-0.019** (-0.034,-0.006)
pMe [c]	8.13%	12.50%	13.57%

Crude model: No adjustments. Model 1: Partially adjusted model, adjusting for age, sex, residence, education level, and marital status. Model 2: Fully adjusted model, additionally adjusting for smoking, drinking, life satisfaction, sleep duration, hypertension, diabetes, lung disease, stroke, mental disorder, heart disease, liver disease, and kidney disease.

[a] Total effects represent the overall impact of the number of children and depression on cognitive functioning, expressed through regression coefficients with 95% confidence intervals (CIs). [b] Indirect effect = Multiplication of Path a and Path b coefficients. Coefficients for indirect effects were determined using the bootstrap method with 1000 repetitions, and a 95%

bias-corrected and accelerated (BCa) confidence interval (CI) was employed to enhance the accuracy of the analysis. [c] pMe is the proportion of the total effect that is mediated. *P < 0.05, **P < 0.01, ***P < 0.001.

Supplementary Table S13. Associations Between Number of Children, Depression, and Cognitive Function in older adults Based on Cross-sectional Study Data (Regrouped)

Models	Episodic Memory		Mental Status		Total Cognition	
	β	*95%CI*	*β*	*95%CI*	*β*	*95%CI*
**Crude model**						
**Number of Children**						
1	Ref.	-	Ref.	-	Ref.	-
0	-0.147*	(-0.282,-0.012)	-0.100*	(-0.192,-0.009)	-0.117**	(-0.204,-0.030)
≥2	-0.111***	(-0.135,-0.087)	-0.082***	(-0.100,-0.065)	-0.093***	(-0.109,-0.076)
**Depression**						
0	Ref.	-	Ref.	-	Ref.	-
1	-0.141***	(-0.166,-0.115)	-0.102***	(-0.119,-0.085)	-0.116***	(-0.132,-0.010)
**Model 1**						
**Number of Children**						
1	Ref.	-	Ref.	-	Ref.	-
0	-0.052	(-0.175,0.071)	-0.051	(-0.135,0.033)	-0.051	(-0.128,0.026)
≥2	-0.014	(-0.038,0.010)	-0.025**	(-0.042,-0.008)	-0.021**	(-0.036,-0.005)
**Depression**						
0	Ref.	-	Ref.	-	Ref.	-
1	-0.106***	(-0.131,-0.083)	-0.062***	(-0.078,-0.046)	-0.078***	(-0.093,-0.063)
**Model 2**						
**Number of Children**						
1	Ref.	-	Ref.	-	Ref.	-
0	-0.059	(-0.180,0.063)	-0.058	(-0.142,0.025)	-0.058	(-0.135,0.018)
≥2	-0.012	(-0.037,0.012)	-0.023**	(-0.040,-0.006)	-0.018*	(-0.034,-0.003)
**Depression**						
0	Ref.	-	Ref.	-	Ref.	-
1	-0.102***	(-0.127,-0.077)	-0.063***	(-0.080,-0.046)	-0.078***	(-0.093,-0.062)

Crude model: No adjustments. Model 1: Partially adjusted model, adjusting for age, sex, residence, education level, and marital status. Model 2: Fully adjusted model, additionally adjusting for smoking, drinking, life satisfaction, sleep duration, hypertension, diabetes, lung disease, stroke, mental disorder, heart disease, liver disease, and kidney disease.

Ref: One child was chosen as the reference value, considering that the participants were affected by the one-child policy in China. *P < 0.05, **P < 0.01, ***P < 0.001
